# Supplementary material for: Cognitive reserve involves decision making and is associated with left parietal and hippocampal hypertrophy in neurodegeneration
Source: Commun Biol. 2024 Jun 18;7:741. doi: 10.1038/s42003-024-06416-x (PMC11189446; doi:10.1038/s42003-024-06416-x)
Supplement: Supplementary file 2 — Supplementary information [file 42003_2024_6416_MOESM2_ESM.pdf]

Supplementary Results

Supplementary Table 1. Demographic and clinical data of participants

	controls	preHDs	earlyHDs
Number of participants	45	20	28
Number of MRI imaging	—	20	26
Sex	26F / 19M	13F / 7M	16F / 12M
Handedness	38R / 7L	19R / 1L	25R / 3L
Age, years[a]	45.4±6.1	41.4±10.3	49.6±11.4
Education, years[a]	13.4±2.1	14.1±2.4	13.1±3.2
Number of CAG repeats[a]	—	43±2.5	45.3±4.8
Total Functional Capacity score[a]	—	13±0	11.7±1
Total Motor Score[a]	—	0.6±1.2	27.5±11.2
Burden score[a]	—	297.1±86.1	444.4±110.2
Time to predicted age-at-onset[a]	—	8.4±10.1	—
Mattis Dementia Rating Scale[a]	—	141.8±2.8	132.9±9.1

preHDs: premanifest participants; earlyHDs: early-stage Huntington's disease patients; number of CAG repeats: pathological threshold > 35; Burden Score= (CAG-35.5) x age; Time to predicted age-at-onset = predicted age-at-onset – actual age, with predicted age-at-onset = 21.54 + exp(9.556 – [0.146 x number of cag repeats]) [8]; Mattis Dementia Rating Scale [61]: pathological score below 136/144; F: female; M: male; R: right-handed; L: left-handed; [a]Data are represented as mean ± standard deviation.

Supplementary Table 2. Cognitive assessment

	Descriptive statistics			Group comparisons		
	controls[a]	preHDs	earlyHDs	preHDs/controls	earlyHDs/controls	preHDs/earlyHDs
Forward digit span	6.4±1	6.4±1.3	5.6±1	[-0.7,0.7], *p*=.994	[-1.4,-0.1], *p*=.016	[0.01,1.5], *p*=.045
Category fluency	33.8±8.1	37.6±7.1	21.8±9.1	[-2.6,9.5], *p*=.373	[-16.7,-5.5], *p*<.001	[8.5,20.7], *p*<.001
Letter fluency	75.6±22.8	68.3±22.9	36.5±16	[-23.1,7.8], *p*=.468	[-51.0,-23.1], *p*<.001	[13.8,45.0], *p*<.001
Stroop color	83.6±11.2	75.7±14	48.6±13.7	[-18.5,1.0], *p*=.089	[-38.9,-21.6], *p*<.001	[11.9,31.3], *p*<.001
Stroop interference	47.5±10.5	47.2±10.2	29.9±12	[-8.9,7.0], *p*=.956	[-21.0,-7.0], *p*<.001	[5.2,20.9], *p*<.001
Stroop word	104.7±16.8	98.4±15.8	66.7±12.3	[-18.4,4.8], *p*=.346	[-45.3,-24.7], *p*<.001	[16.7,39.7], *p*<.001
Symbol digit modalities Test	49.5±7.7	54±11.1	28.7±7.8	[-3.5,9.9], *p*=.497	[-23.7,-11.4], *p*<.001	[14.1,27.3], *p*<.001
TMT A RT	25±6.8	25.7±10.5	54.3±16.9	[-8.2,10.8], *p*=.946	[17.4,34.4], *p*<.001	[-34.1,-15.1], *p*<.001
TMT A correct	24.7±0.5	24.9±0.3	25±0	[-0.1,0.5], *p*=.211	[0.02,0.5], *p*=.032	[-0.3,0.2], *p*=.820
TMT B RT	63.6±22.2	69.8±34	164.7±62.3	[-26.4,41.3], *p*=.858	[63.8,124.3], *p*<.001	[-120.4,-52.8], *p*<.001
TMT B correct	24.7±0.6	24.6±0.8	22.3±3.9	[-1.9,1.7], *p*=.995	[-3.8,-0.6], *p*<.01	[0.3,4.0], *p*=.0173

Descriptive statistics (mean±standard deviation) of the three groups tested with forward digit span, category fluency, Unified Huntington's Disease Rating scale cognitive scores, and Trail Making Test (TMT). Group comparisons: Tukey's post-hoc test [95% confidence interval] and the *p*-value of each pair comparison are reported only if there was a significant main effect of group. preHDs: premanifest participants; earlyHDs: early-stage Huntington's disease patients; [a] Cohort of controls restricted to 27 participants.

Supplementary Table 3. Behavioral performances and DDM parameters

	Descriptive statistics			Group comparisons		
	controls	preHDs	earlyHDs	preHDs/controls	earlyHDs/controls	preHDs/earlyHDs
Accuracy	0.98±0.02	0.98±0.02	0.93±0.04	[-0.021,0.016], *p*=.96	[-0.059,-0.026], *p*<.001	[0.021,0.061], *p*<.001
Response time	0.6±0.19	0.55±0.14	1.04±0.28	[-0.176,0.099], *p*=.78	[0.303,0.549], *p*<.001	[-0.615,-0.315], *p*<.001
Response threshold, a	2.37±0.73	2.8±1.00	3.02±1.18	[-0.19,1.1], *p*=.24	[0.15,1.22], *p*<.01	[-0.93,0.38], *p*=.57
Drift rate, v	2.64±0.82	3.13±0.96	1.54±0.47	[-0.078,0.93], *p*=.11	[-1.48,-0.58], *p*<.001	[0.91,2.01], *p*<.001
Non-decision time, Ter	0.07±0.07	0.07±0.06	0.08±0.10	_	_	_
Bias, zr	0.49±0.03	0.49±0.02	0.49±0.04	_	_	_

Mean accuracy, mean response time, mean response threshold, mean drift rate, mean non-decision time and mean Bias of participants in the auditory language discrimination task. Descriptive statistics (mean±standard deviation) of each group. Group comparisons: Tukey's post-hoc test [95% confidence interval]) and *p*-value of each pair comparison are displayed only if ANOVA showed significant main effect of group. preHDs: premanifest participants; earlyHDs: early-stage Huntington's disease patients.

Supplementary Table 4. Neuroanatomical sub-cortical differences between groups

	Descriptive statistics			Group comparisons		
	controls	preHDs	earlyHDs	preHDs/controls	earlyHDs/controls	preHDs/earlyHDs
striatum	.0113±0 .001	.0099± .0018	.0075± .0011	[.0010, .0019], *p*<.0001	[- .0043, - .0034], *p*<.0001	[- .0029, - .0019], *p*<.0001
pallidum	.0025± .0003	.0023± .0004	.0017± .0002	[- .0002, .0007], *p*=.39	[- .0012, - .0003], *p*<.001	[- .0010, .0000], *p*=.0510
thalamus	.0093± .0008	.0094±.0 007	.0087± .0005	[- .0005, .0004], *p*=.96	[- .0010, - .0001], *p*<.01	[- .0011 ,- .0001], *p*<.01
hippocampus	.0050± .0004	.0051±.0 004	.0047± .0005	[- .0006, .0004], *p*=.83	[- .0006, .0002], *p*=.42	[- .0008, .0001], *p*=.21
amygdala	.0019± .0002	.0019±.0 003	.0018± .0002	[- .0004, .0005], *p*=.96	[- .0006, .0003], *p*=.82	[- .0006, .0004], *p*=.96

Mean subcortical volumes. Descriptive statistics (mean±standard deviation) of each group. Group comparisons: Tukey's post-hoc test [95% confidence interval]) and *p*-value of each pair comparison corrected for the three multiple comparisons. preHDs: premanifest participants; earlyHDs: early-stage Huntington's disease patients.

Supplementary Table 5. Cortical thickness differences between groups

Cortical region	Cluster size (mm^2)	MNI coordinates x	y	z	Cluster-wise *p*-value
EarlyHDs < imaging controls					
L angular gyrus	68.16	-29	-50	37	0.02050
L occipital superior	206.24	-23	-85	27	0.00020
R lateral occipital	63.40	28	-83	14	0.02560
R caudal middle frontal	76.32	39	21	45	0.01600
EarlyHDs < preHDs					
R lateral occipital	102.15	31	-87	13	0.00610

L: left; R: right; preHDs: premanifest participants; earlyHDs: early-stage Huntington's disease patients.

Supplementary Table 6. Drift rate according to age onset

	Descriptive statistics	Group comparisons				
		controls	far preHDs	middle preHDs	close preHDs	earlyHDs
controls	2.64±0.82	–	[-0.99,0.71], *p*=.99	[0.26,1.79], *p*<.01	[-1.09,1.10], *p*=1	[-1.53,-0.52], *p*<.001
far preHDs	2.65±0.87		–	[0.11, 2.22], p<.05	[-1.45,1.18], *p*= .99	[0.004, 1.78], *p*<.05
middle preHDs	3.72±0.79			–	[-0.23,2.29], *p*=.16	[1.25,2.86], *p*<.001
close preHDs	2.65±0.87				–	[-2.15,0.09], *p*= 0.09
earlyHDs	1.54±0.47					—

Mean drift rate of participants according to to time to predicted age-at-onset. Descriptive statistics (mean±standard deviation) of each group. Group comparisons: Tukey's post-hoc test [95% confidence interval]) and *p*-value of each pair comparison. preHDs: premanifest participants; earlyHDs: early-stage Huntington's disease patients.

Supplementary Table 7. Effect of response threshold on the drift rate

Descriptive statistics			Group comparisons		
controls	preHDs	earlyHDs	preHDs/controls	earlyHDs/controls	preHDs/earlyHDs
0.28±0.14	0.71±0.15	0.28±0.11	[-0.12,0.99], *p*<.05	[-0.45,0.47], *p*=.97	[-0.93,0.07], *p*<.05

Mean response threshold and mean drift rate. Descriptive statistics (mean±standard deviation) of each group. Group comparisons: Tukey's post-hoc test [95% confidence interval] and *p*-value of each group comparison when there was a significant group effect. preHDs: premanifest participants; earlyHDs: early-stage Huntington's disease patients.

Supplementary Table 8. Relationships between cortical thickness, and accuracy and response time

Cortical region	Cluster size (mm^2)	MNI coordinates (x	y	z)	Cluster-wise p-value
Accuracy					
L superior parietal	73.48	-29	-46	59	0.03803
Response time					
L lateral occipital	180.40	-33	-92	-9	0.00100
L superior parietal	103.93	-24	-75	25	0.01157
L precuneus	80.38	-20	-71	22	0.02780
L lateral occipital *	136.25	-33	-86	8	0.00459
L superior parietal *	96.43	-24	-59	56	0.01832
R occipital	79.84	30	-70	21	0.02780
R superior occipital *	68.50	10	-82	34	0.04645

L: left; R: right; *: significant for one-tail test only (red clusters in Supplementary Fig. 2).

Supplementary Table 9. Model selection

	Observed	Full model	Parsimonious model
Group distribution	—	a, v, zr, Ter	a, v
Deviance information criterion	—	12412	12410
Proportion of "same" responses	0.51	0.5±0.48; 0.23	0.5±0.48; 0.23
Response time distribution for "different" responses			
Mean	0.85	0.70±0.34; 0.14	0.70±0.35; 0.14
10th	0.27	0.39±0.23; 0.07	0.39±0.24; 0.07
30th	0.41	0.50±0.26; 0.08	0.50±0.26; 0.08
50th	0.57	0.62±0.31; 0.10	0.62±0.31; 0.10
70th	0.83	0.79±0.39; 0.16	0.79±0.4; 0.16
90th	1.49	1.10±0.60; 0.51	1.10±0.60; 0.51
Response time distribution for "same" responses			
Mean	0.74	0.67±0.34; 0.12	0.67±0.34; 0.12
10th	0.26	0.37±0.23; 0.07	0.37±0.23; 0.06
30th	0.39	0.48±0.26; 0.08	0.48±0.26; 0.07
50th	0.53	0.60±0.31; 0.10	0.60±0.31; 0.10
70th	0.76	0.76±0.39; 0.16	0.76±0.39; 0.15
90th	1.41	1.07±0.60; 0.48	1.07±0.60; 0.48

For each model, the parameters with group distributions are reported: a is the response threshold, v the drift rate, zr the relative bias, and Ter the non-decision time. The deviance information criterion is reported for each model. Posterior predictive check summary statistics for the data simulated with the full and parsimonious models are also reported. For models, mean ±standard deviation/ mean square error are reported for the mean response time and the 10th, 30th, 50th, 70th, and 90th percentiles of the response time distributions for each response ("same" and "different"). The values reported for the two models are to compare with the values of the observed data.

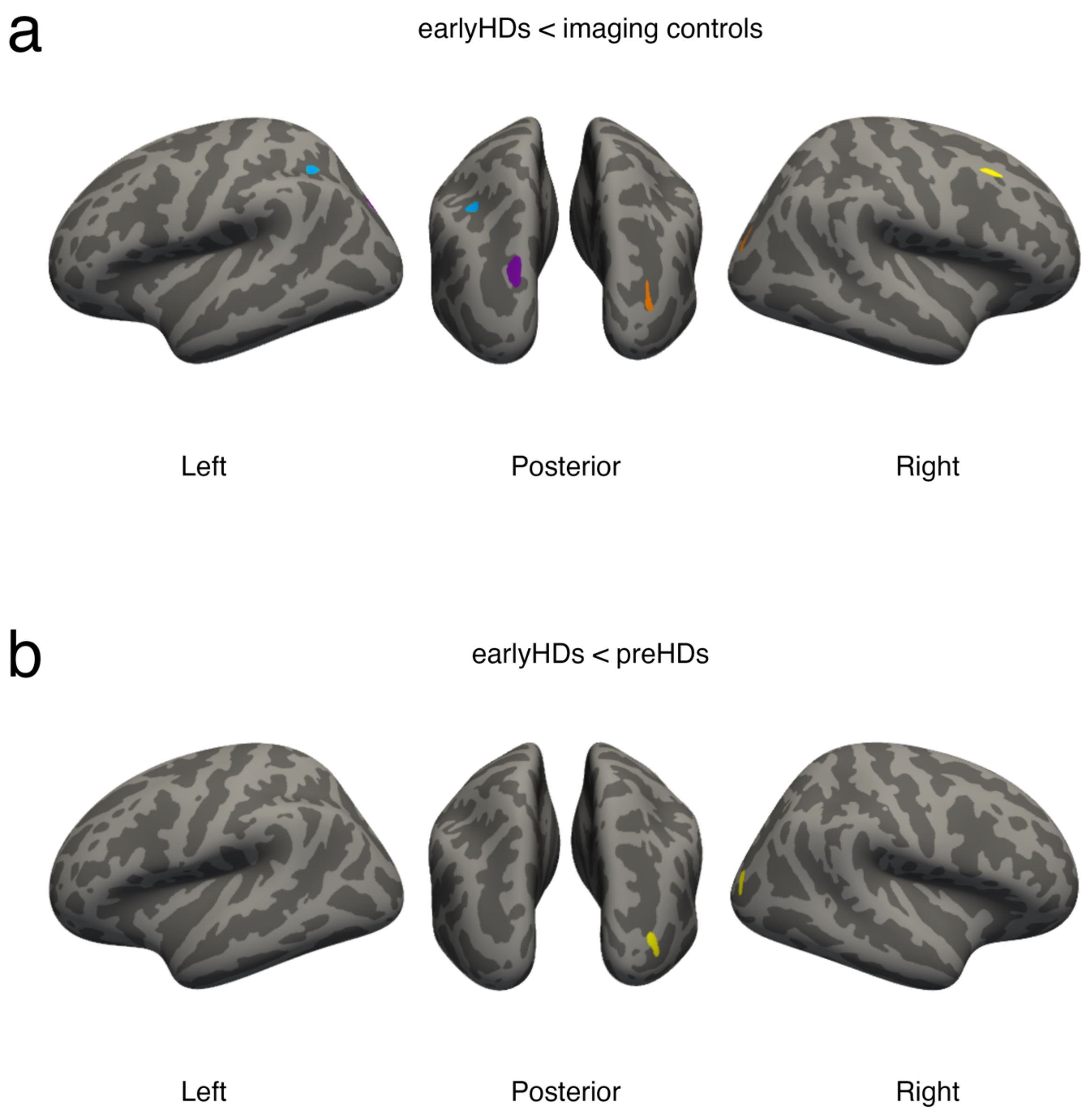

Supplementary Figure 1. Neuroanatomical differences between groups
(a) Cortical maps showing significant cortex thinning in earlyHDs as compared with imaging controls. Each cluster is represented in a different color: blue: left angular gyrus, purple: left occipital superior, orange: right lateral occipital, and yellow: right caudal middle frontal. (b) Cortical maps of differences between earlyHDs and preHDs showing significant thinner cortex in earlyHDs compared with that of preHDs. Yellow represents right lateral occipital, light grey represents gyrus, and dark grey represents sulcus.

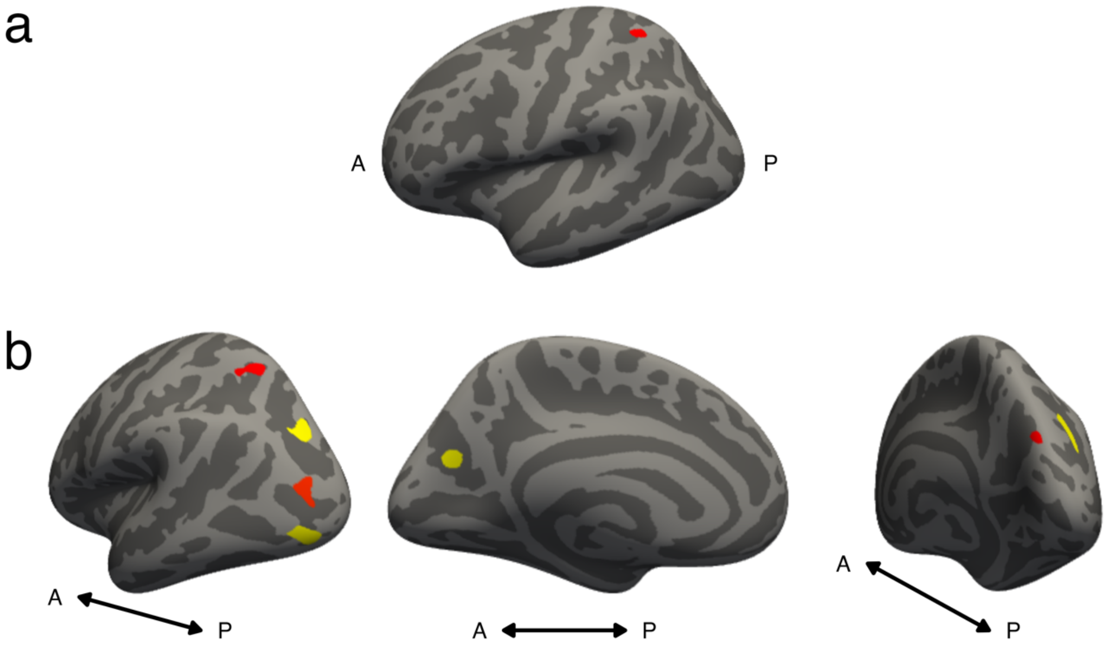

Supplementary Figure 2. Relationship between cortical thickness, and accuracy and response time

Cortical maps of significant clusters with (a) accuracy (positive relationship) and (b) response times (negative relationship). Light grey represents gyrus and dark grey represents sulcus. Red: significant clusters for one-tailed test, Yellow: significant clusters for two-tailed test. A: Anterior, P: Posterior.

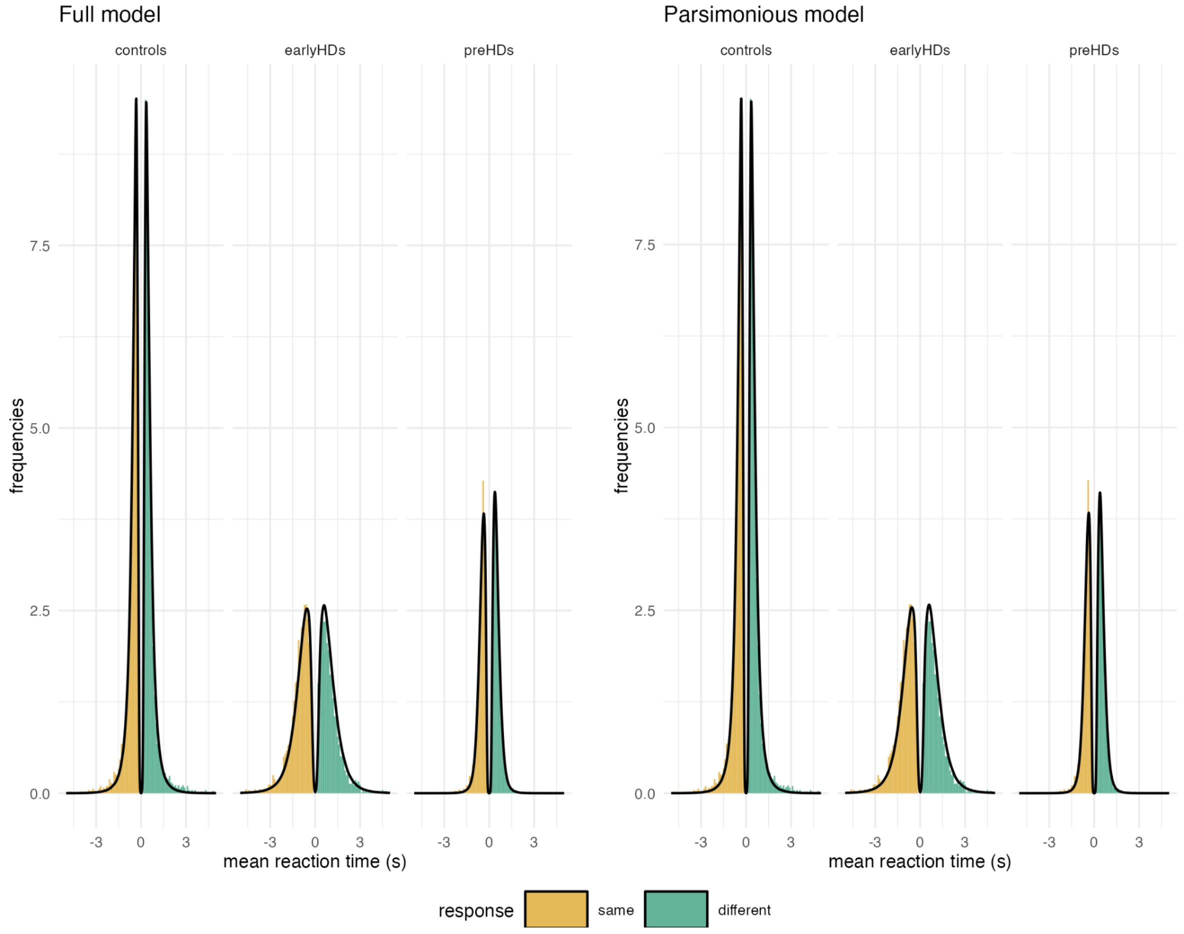

Supplementary Figure 3. Posterior predictive checks for the full and parsimonious models
The distribution along the positive x-axis shows the reaction time distribution for "different" trials in green while the distribution along the negative x-axis shows the reaction time distribution for "same" trials in yellow. Each panel shows the normalized histograms of the observed data and the model prediction (solid black line) for each group (controls, preHDs and earlyHDs).
