# Supplementary material for: Cognitive reserve involves decision making and is associated with left parietal and hippocampal hypertrophy in neurodegeneration
Source: Commun Biol. 2024 Jun 18;7:741. doi: 10.1038/s42003-024-06416-x (PMC11189446; doi:10.1038/s42003-024-06416-x)
Supplement: Supplementary file 3 — Reporting summary [file 42003_2024_6416_MOESM3_ESM.pdf]

nature portfolio

Corresponding author(s): Charlotte Jacquemot

Last updated by author(s): May 14, 2024

Reporting Summary

Nature Portfolio wishes to improve the reproducibility of the work that we publish. This form provides structure for consistency and transparency in reporting. For further information on Nature Portfolio policies, see our Editorial Policies and the Editorial Policy Checklist.

Statistics

For all statistical analyses, confirm that the following items are present in the figure legend, table legend, main text, or Methods section.

n/a	Confirmed	
☐	☒	The exact sample size (*n*) for each experimental group/condition, given as a discrete number and unit of measurement
☐	☒	A statement on whether measurements were taken from distinct samples or whether the same sample was measured repeatedly
☐	☒	The statistical test(s) used AND whether they are one- or two-sided<br>*Only common tests should be described solely by name; describe more complex techniques in the Methods section.*
☐	☒	A description of all covariates tested
☐	☒	A description of any assumptions or corrections, such as tests of normality and adjustment for multiple comparisons
☐	☒	A full description of the statistical parameters including central tendency (e.g. means) or other basic estimates (e.g. regression coefficient) AND variation (e.g. standard deviation) or associated estimates of uncertainty (e.g. confidence intervals)
☐	☒	For null hypothesis testing, the test statistic (e.g. *F*, *t*, *r*) with confidence intervals, effect sizes, degrees of freedom and *P* value noted<br>*Give P values as exact values whenever suitable.*
☐	☒	For Bayesian analysis, information on the choice of priors and Markov chain Monte Carlo settings
☐	☒	For hierarchical and complex designs, identification of the appropriate level for tests and full reporting of outcomes
☐	☒	Estimates of effect sizes (e.g. Cohen's *d*, Pearson's *r*), indicating how they were calculated

Our web collection on statistics for biologists contains articles on many of the points above.

Software and code

Policy information about availability of computer code

Data collection	PsyScope X was used for data collection. This sofware is no longer updated.<br>http://psy.cns.sissa.it/<br>Cohen, Jonathan; MacWhinney, Brian; Flatt, Matthew; Provost, Jefferson (June 1993). "PsyScope: A new graphic interactive environment for designing psychology experiments". Behavior Research Methods, Instruments, and Computers 25 (2): 257–271. doi:10.3758/BF03204507.
Data analysis	Freesurfer (6) , R (4.2.3) , HDDM (0.6.0)

For manuscripts utilizing custom algorithms or software that are central to the research but not yet described in published literature, software must be made available to editors and reviewers. We strongly encourage code deposition in a community repository (e.g. GitHub). See the Nature Portfolio guidelines for submitting code & software for further information.

Data

Policy information about availability of data

All manuscripts must include a data availability statement. This statement should provide the following information, where applicable:

- Accession codes, unique identifiers, or web links for publicly available datasets
- A description of any restrictions on data availability
- For clinical datasets or third party data, please ensure that the statement adheres to our policy

All participants signed an informed consent form guaranteeing data confidentiality. The conditions of our ethics approval, including the ethical consent by

participants, do not permit public archiving of anonymized study data. Readers seeking access to the data should contact Prof. Anne-Catherine Bachoud-Lévi. Access will be granted to named individuals in accordance with ethical procedures governing the reuse of sensitive data, including a research partnership and the completion of a data transfer agreement provided by the APHP. Legal copyright restrictions prevent public archiving of the UHDRS, which can be obtained from UHDRS® | - Huntington Study Group.

Research involving human participants, their data, or biological material

Policy information about studies with human participants or human data. See also policy information about sex, gender (identity/presentation), and sexual orientation and race, ethnicity and racism.

Reporting on sex and gender	Because of the genetic tranmission of Huntington Disease, only sex was considered (and not gender). Sex was determined based on self-reporting. Consent has been obtained for collecting this data.
Reporting on race, ethnicity, or other socially relevant groupings	/
Population characteristics	Age, Laterality and Education was considered to control for group matching between Healthy controls, Premanifest gene carriers and Huntington Disease patients and were considered as covariate-relevant population characteristic of the human research participants.<br>Number of CAG repeats, MDRS (Mattis Dementia Rating Scale), UHDRS (Unified HuntingtonDisease Rating Scale, international battery for assessing Huntington Disease gene carriers) were used to define the three groups of subjects (Healthy controls, Premanifest gene carriers and Huntington Disease Patients).
Recruitment	Participants were recruited from an outpatient clinical biomarker study (NCT01412125).
Ethics oversight	Ethics committee of Henri Mondor Hospital (Créteil, France).

Note that full information on the approval of the study protocol must also be provided in the manuscript.

Field-specific reporting

Please select the one below that is the best fit for your research. If you are not sure, read the appropriate sections before making your selection.

☐ Life sciences ☒ Behavioural & social sciences ☐ Ecological, evolutionary & environmental sciences

For a reference copy of the document with all sections, see nature.com/documents/nr-reporting-summary-flat.pdf

Behavioural & social sciences study design

All studies must disclose on these points even when the disclosure is negative.

Study description	Quantitative data, cross-sectional data
Research sample	HD patients, premanifest
Sampling strategy	The sample size was estimated on the basis of previous work (Teichmann et al., 2009). The inclusion phase of the studied ended when 45 valid brain MRI scans had been obtained from mutation carriers.<br>Teichmann, M., Darcy, I., Bachoud-Lévi, A.-C., Dupoux, E., 2009. The role of the striatum in phonological processing. Evidence from early stages of Huntington's disease. Cortex 45, 839–849. https://doi.org/10.1016/j.cortex.2008.12.005
Data collection	Clinical data were collected using paper-and-pencil tests.<br><br>Experimental data from the language discrimination task, was collected with a computer. The participants were asked to sit in front of an Apple MacBook Pro, in a quiet room, wearing headphones tuned to ensure hearing comfort and had to press P for "pareil" (the French word for "same") or D for "different", on an AZERTY keyboard. Participants were informed that their accuracy and response time would be recorded, and were advised to answer as accurately and quickly as possible. An experimenter was present besides the participant, and was blind to experimental condition during data collection.<br><br>Three-dimensional T1-weighted structural MRI scans were acquired with an MP-RAGE sequence on a Siemens symphony 1.5 Tesla whole-body scanner (Henri Mondor Hospital, Paris, France)
Timing	Participants were recruited between December 2013 and July 2017
Data exclusions	No participants were excluded as they were screen beforehand for inclusion criteria.
Non-participation	No participants declined to participate.
Randomization	Participants were allocated into experimental groups due to their genetic status and developpement of the disease. We measured

Randomization	cofounding variables such as sex, education and handedness and controlled post hoc that the three groups were matched. Age was introduced as a covariate in analyses to control for age difference between the two experimental groups due to the developpement of the disease.

Reporting for specific materials, systems and methods

We require information from authors about some types of materials, experimental systems and methods used in many studies. Here, indicate whether each material, system or method listed is relevant to your study. If you are not sure if a list item applies to your research, read the appropriate section before selecting a response.

Materials & experimental systems

n/a	Involved in the study
☒	☐ Antibodies
☒	☐ Eukaryotic cell lines
☒	☐ Palaeontology and archaeology
☒	☐ Animals and other organisms
☒	☐ Clinical data
☒	☐ Dual use research of concern
☒	☐ Plants

Methods

n/a	Involved in the study
☒	☐ ChIP-seq
☒	☐ Flow cytometry
☐	☒ MRI-based neuroimaging

Plants

Seed stocks	*Report on the source of all seed stocks or other plant material used. If applicable, state the seed stock centre and catalogue number. If plant specimens were collected from the field, describe the collection location, date and sampling procedures.*
Novel plant genotypes	*Describe the methods by which all novel plant genotypes were produced. This includes those generated by transgenic approaches, gene editing, chemical/radiation-based mutagenesis and hybridization. For transgenic lines, describe the transformation method, the number of independent lines analyzed and the generation upon which experiments were performed. For gene-edited lines, describe the editor used, the endogenous sequence targeted for editing, the targeting guide RNA sequence (if applicable) and how the editor was applied.*
Authentication	*Describe any authentication procedures for each seed stock used or novel genotype generated. Describe any experiments used to assess the effect of a mutation and, where applicable, how potential secondary effects (e.g. second site T-DNA insertions, mosiacism, off-target gene editing) were examined.*

Magnetic resonance imaging

Experimental design

Design type	Anatomical
Design specifications	One Sequence
Behavioral performance measures	None

Acquisition

Imaging type(s)	Anatomical
Field strength	1,5
Sequence & imaging parameters	Three-dimensional, T1-weighted structural scans were acquired with a MP-RAGE sequence on a Siemens symphony 1.5 Tesla whole-body scanner (Henri Mondor Hospital, Paris, France) with a 12-channel head coil (TR=2400 ms, TE=3.72 ms, TI=1000 ms, FA=8°, FOV=256*256 mm2, 1-mm isotropic voxel, slice thickness=1 mm, no inter-slice gap, 160 sagittal sections).
Area of acquisition	Whole brain scan

Diffusion MRI ☐ Used ☒ Not used

Preprocessing

Preprocessing software	MRI scans were preprocessed with Freesurfer (version 6) (http://surfer.nmr.mgh.harvard.edu/). The procedure included the removal of non-brain tissue, normalization of the intensity of the grey/white matter boundary, automated topology correction, and surface deformation. The following subcortical structures were automatically segmented: thalamus, striatum, pallidum, hippocampus, and amygdala. Cortical thickness (in mm) was calculated as the shortest distance between the grey/

white matter boundary and the pial surface at each vertex across the cortical mantle. All reconstructed data were visually checked for segmentation accuracy by a neuropsychologist (ML) trained in brain structural segmentation analysis, and reviewed by an expert neurologist blinded to participants' genetic makeup.

Normalization

The spherical cortical thickness data of all participants were mapped onto an "average" subject by surface-based registration methods (Fischl, B., Sereno, M., and Dale, A. (1999). Cortical surface-based analysis. Neuroimage 9, 195–207.) to morphologically match homologous cortical locations in participants. We used a 10-mm full width at half-maximum Gaussian kernel to smooth maps of cortical thickness.

Normalization template

Due to the specificity of our population, data were normalized to an homemade template based on an the average of our subjects in our cohort.

Noise and artifact removal

The procedure included the removal of non-brain tissue, normalization of the intensity of the grey/white matter boundary, automated topology correction, and surface deformation.

Volume censoring

Define your software and/or method and criteria for volume censoring, and state the extent of such censoring.

Statistical modeling & inference

Model type and settings

Neuroanatomical differences between groups
Vertex-wise comparisons of cortical thickness values between groups were performed on Freesurfer using generalized linear models, with cortical thickness as the dependent variable, group as the predictive factor, and age as a covariate.

Relationship between brain structure and DDM parameters in mutation carriers
we fitted a generalized linear model for each parameter with the cortical thickness as the dependent variable, the parameter and the disease stage as predictive variables, and age as a covariate.

Effect(s) tested

Neuroanatomical differences between groups
We tested the three groups comparisons.

Relationship between brain structure and DDM parameters in mutation carriers
We tested the hypothesis of an interaction between cortical thickness and disease stage. If there was no cluster with a significant interaction, the disease stage was removed from the analysis before testing the hypothesis of the non-null relationship (two-tailed test) between the DDMs parameter and the cortical thickness.

Specify type of analysis: ☐ Whole brain ☐ ROI-based ☒ Both

Anatomical location(s)

we used the clusters identified by the generalized linear model analyses in mutation carriers as regions of interest from which we extracted cortical thickness values for imaging controls and mutation carriers.

Statistic type for inference
(See Eklund et al. 2016)

Vertex wise

Correction

At each vertex, F-statistics were calculated to test the hypothesis (two-tailed test). We corrected for multiple comparisons by family-wise error cluster-based correction, using Monte Carlo simulations with 10,000 iterations.

Models & analysis

n/a	Involved in the study
☒	☐ Functional and/or effective connectivity
☒	☐ Graph analysis
☒	☐ Multivariate modeling or predictive analysis
